# Supplementary figures and images for: Exploring the metabolic profile of A. baumannii for antimicrobial development using genome-scale modeling
Source: PLoS Pathog. 2024 Sep 23;20(9):e1012528. doi: 10.1371/journal.ppat.1012528 (PMC11463759; doi:10.1371/journal.ppat.1012528)

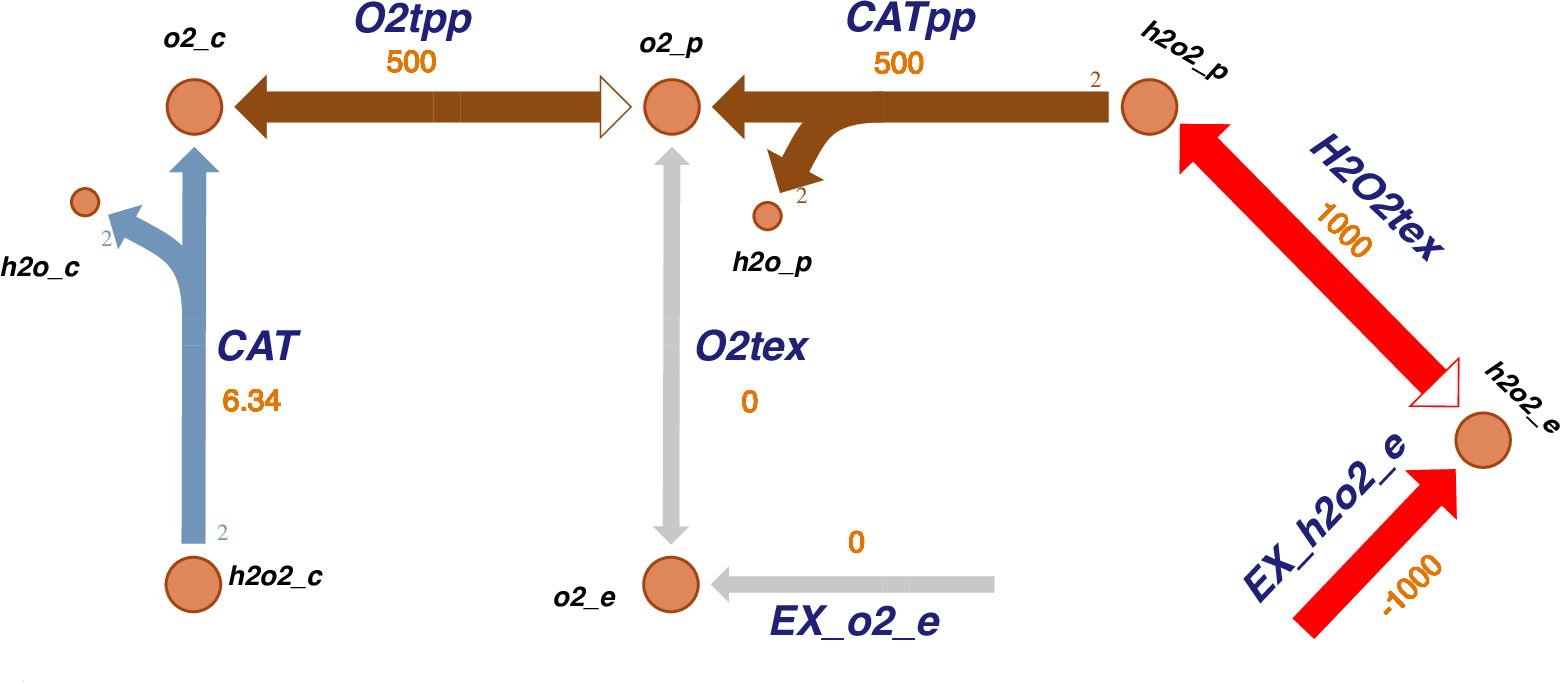

Supplement: S1 Fig — All flux rates are written in orange and are given in mmol/(gDW · h). The reaction abbreviations are as follows: O2tpp, O2 transport via diffusion between periplasm and cytosol; CATpp, periplasmatic catalase; H2O2tex, hydrogen peroxide transport via diffusion; CAT, catalase; O2tex, O2 transport via diffusion between periplasm and extracellular space; EX_h2o2_e, hydrogen peroxide exchange and EX_o2_e, O2 exchange. Figure generated with Escher [109]. (TIF) [file ppat.1012528.s001.tif]

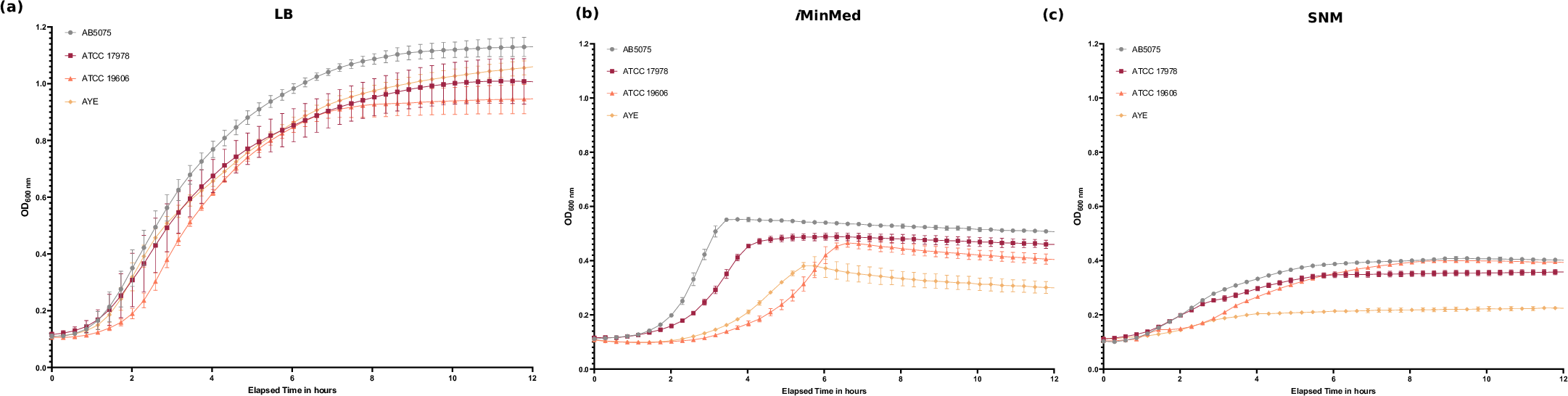

Supplement: S2 Fig — The growth curves for A. baumannii strains AB5075, ATCC 73217978, ATCC 19606, and AYE were measured in LB and SNM. Additionally, the in silico-defined minimal medium (iMinMed) was tested for all strains. (TIF) [file ppat.1012528.s002.tif]

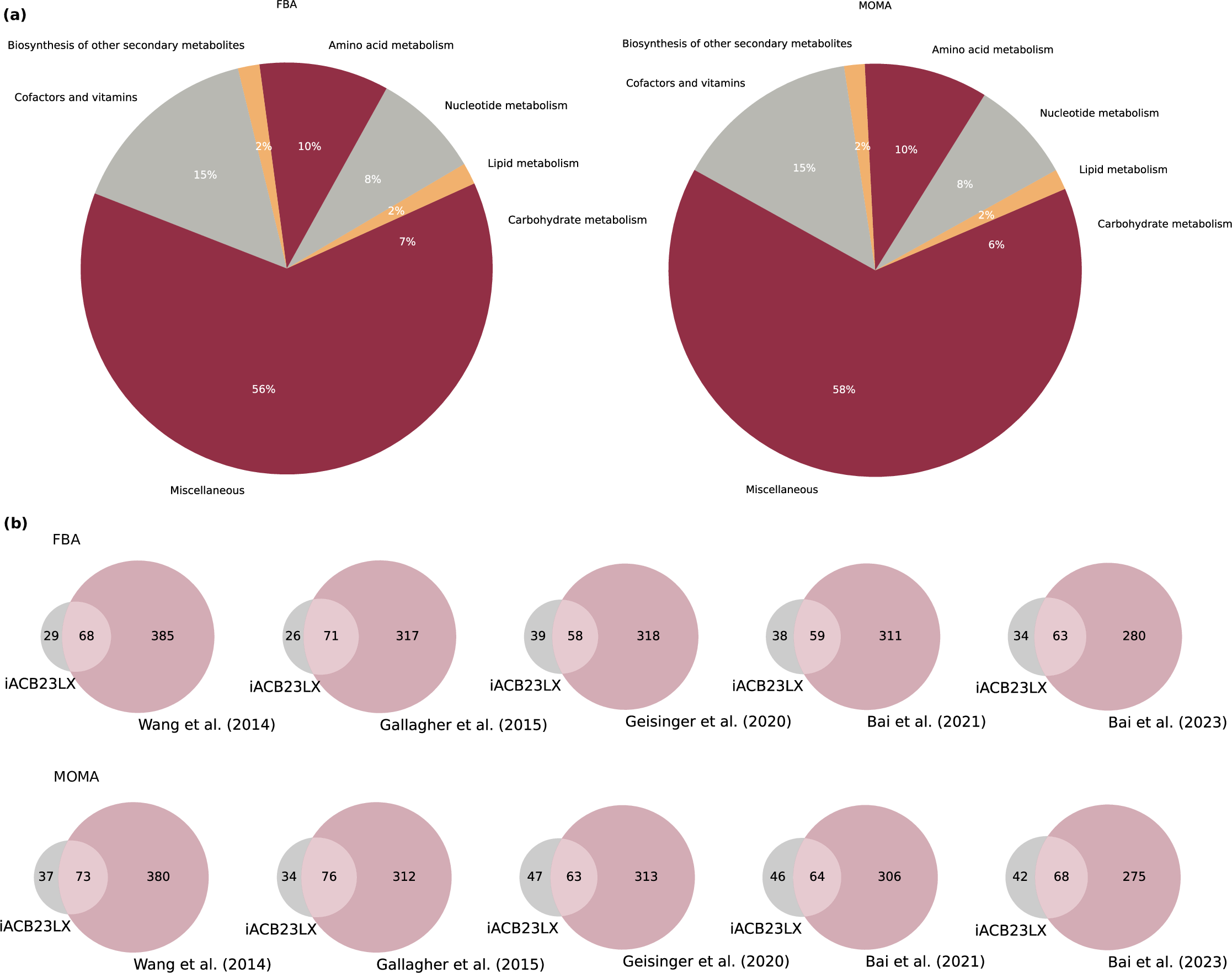

Supplement: S3 Fig — (a) Metabolic subsystems distribution of all essential genes reported in various Tn-seq studies and predicted using iACB23LX (true negatives). (b) Venn diagrams of essential genes from five examined Tn-seq datasets compared to essential genes predicted by the model developed in this study. (TIF) [file ppat.1012528.s003.tif]
